# Lamellar Dilation in (AB)-g-A Copolymacromer Melts: A Dissipative Particle Dynamics Study

**DOI:** 10.3390/polym18070798

**Published:** 2026-03-26

**Authors:** Jihoon Park, June Huh

**Affiliations:** Department of Chemical and Biological Engineering, Korea University, Seoul 02841, Republic of Korea; ayanami9306@korea.ac.kr

**Keywords:** copolymacromer, hybrid bottlebrush copolymer, block copolymer/homopolymer blends, microphase separation, macrophase separation, microphase dilation, lamellar swelling, dissipative particle dynamics, phase diagram, tethered homopolymer

## Abstract

Homopolymer addition is a widely used strategy to dilate the microdomain spacing of block copolymers, yet the attainable dilation is often limited by macrophase separation in conventional blends at elevated homopolymer loading. In this work, we investigate an architectural route to suppress macrophase separation while retaining homopolymer-driven dilation: a covalently hybridized bottlebrush copolymer (CH-BBC), a copolymacromer-like bottlebrush architecture in which symmetric AB diblock side chains and A-type homopolymer side chains are covalently grafted to a common backbone. Using dissipative particle dynamics (DPD) simulations, we directly compare the phase behavior of CH-BBC melts with that of composition-matched blends of symmetric AB diblocks and A-type homopolymers. Across the explored window, CH-BBC exhibits microphase morphologies and disorder without an observable two-phase region, whereas the corresponding blends show extensive two-phase coexistence at elevated homopolymer loading. Lamellar analysis and one-dimensional density decompositions further reveal that CH-BBC enables substantially larger microphase dilation and stronger selective swelling of the A-rich domain because tethered A-type homopolymer segments preferentially occupy and dilate the A-rich domain interior while diblock A segments remain localized near interfaces.

## 1. Introduction

Block copolymers (BCPs) spontaneously form periodic nanostructures in the melt through microphase separation, enabling access to lamellae, cylinders, gyroid-like bicontinuous networks, and spherical micelles with characteristic periods typically in the 5–100 nm range. Because the domain spacing and morphology are directly linked to molecular parameters, BCP self-assembly has become a key design platform for nanopatterning [1,2,3,4,5], photonic materials [6,7,8,9], ion transport media [10,11,12,13], and templated nanofabrication [14,15,16]. A recurring practical objective is to tune the domain spacing (or selectively swell one domain) while maintaining a single, well-defined microphase-separated morphology. Importantly, the preferred direction of this tuning is application-dependent: semiconductor nanopatterning typically drives BCPs toward a smaller pitch, whereas photonic, ion transport, and templated architectures often benefit from a larger pitch or selective domain swelling.

A classical strategy to enlarge the microdomain spacing is to add a homopolymer that is chemically identical to one block [17,18,19]. In the microphase-separated state, such a homopolymer can preferentially partition into the corresponding microdomain and act as a diluent, thereby increasing the domain size and the overall period. This approach is conceptually simple and experimentally accessible, but it is frequently limited by macrophase separation in conventional blends [20,21,22,23,24]. At sufficiently high homopolymer loading, the system may undergo macroscopic demixing into a homopolymer-rich phase and a BCP-rich phase, preempting further controlled microphase dilation and complicating both processing and structural fidelity. In this sense, macrophase separation constitutes a fundamental bottleneck that restricts the attainable range of microphase dilation in blend-based formulations [25,26,27].

Architectural design offers an alternative pathway to decouple microphase dilation from macrophase demixing [2,28]. In comb and bottlebrush copolymers, densely grafted side chains and backbone connectivity modify conformational entropy and correlation lengths, often shifting phase boundaries relative to linear analogs and enabling unconventional scaling of periodicity with architectural parameters [29,30,31,32,33]. More broadly, the connectivity constraints inherent to grafted architectures can suppress demixing pathways available to free chains in blends, suggesting that homopolymer-driven dilation may be realized more robustly when the homopolymer is incorporated into the microphase-forming scaffold rather than introduced as a separate free component.

**Figure 1:**
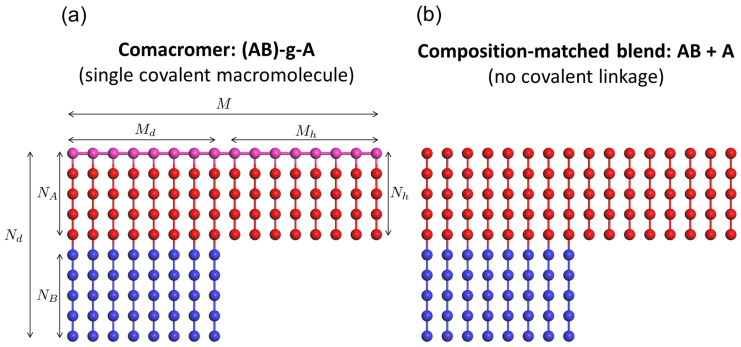
Model definition and architectural comparison. (**a**) Copolymacromer-like hybrid bottlebrush, denoted as (AB)-g-A, in which $M_{d}$ symmetric AB diblock side chains and $M_{h}$ A-type homopolymer side chains are covalently grafted onto a common linear backbone, forming a single macromolecule. The total backbone length is denoted by *M* $\left(=M_{d}+M_{h}\right)$. The backbone beads are shown in pink (chemically identical to A segments) to highlight covalent connectivity. The symmetric diblock consists of $N_{A}$ and $N_{B}$ segments, with total length $N_{d}=N_{A}+N_{B}$, while the homopolymer side chain has length $N_{h}$. (**b**) Composition-matched blend of symmetric AB diblock chains and A-type homopolymer chains (AB + A) without covalent linkage between the two species. Red and blue beads represent A and B segments, respectively.

Here we examine this idea using a copolymacromer-like hybrid bottlebrush architecture in which symmetric AB diblock side chains and A-type homopolymer side chains are covalently grafted onto a common linear backbone (Figure 1a). We refer to this architecture as a covalently hybridized bottlebrush copolymer (CH-BBC). Because CH-BBC is modeled as a one-component melt in which AB and A segments are covalently integrated within the same macromolecular scaffold, blend-like macrophase demixing is excluded by construction. The central question is therefore how far microphase dilation—and in particular lamellar dilation—can be driven by increasing the tethered A-type homopolymer content while retaining an ordered, single-microphase morphology and how this accessible dilation window compares with that of composition-matched blends of free symmetric AB diblocks and free A-type homopolymers (Figure 1b), where lamellar stability and phase behavior are strongly constrained by the presence of free homopolymers.

Using dissipative particle dynamics (DPD) simulations, we map the phase behavior as a function of the homopolymer segment fraction ϕ and the side-chain length ratio $\alpha \equiv N_{h}/N_{d}$, where $N_{d}$ and $N_{h}$ denote the lengths of the symmetric AB diblock and A-type homopolymer side chains, respectively (see Figure 1). We then quantify microphase dilation through the normalized domain spacing $L/L_{0}$, where *L* is the lamellar period and $L_{0}$ is the reference lamellar period at ϕ=0, and analyze selective domain swelling via the lamellar width ratio $L_{A}/L_{B}$, where $L_{A}$ and $L_{B}$ are the thicknesses of the A- and B-rich domains, respectively, under strong segregation.

## 2. Simulation Methods

All simulations were performed using dissipative particle dynamics (DPD) in the standard bead–spring formulation [34,35,36]. The simulation protocol and parameter values are identical to those used in our previous study on bottlebrush copolymers [37], except where otherwise specified. In DPD, the equation of motion for bead *i* with position $r_{i}$ and velocity $v_{i}$ is written as the sum of pairwise-additive nonbonded forces and bonded spring forces:

(1) $$m\frac{d^{2}r_{i}}{dt^{2}}=\sum_{j\neq i}\left(F_{ij}^{\left(C\right)}+F_{ij}^{\left(D\right)}+F_{ij}^{\left(R\right)}+F_{ij}^{\left(S\right)}\right),$$

where $F_{ij}^{\left(C\right)}$, $F_{ij}^{\left(D\right)}$, $F_{ij}^{\left(R\right)}$, and $F_{ij}^{\left(S\right)}$ denote the conservative, dissipative, random, and spring forces between beads *i* and *j*, respectively. The conservative force is a soft repulsion truncated at the cutoff distance $r_{c}$,

(2) $$F_{ij}^{\left(C\right)}=\left\{\begin{array}{cc} a_{ij}\left(1-\frac{r_{ij}}{r_{c}}\right){\hat{r}}_{ij}, & r_{ij}<r_{c},\\ 0, & r_{ij}\geq r_{c}, \end{array}\right.$$

where $a_{ij}$ is the maximum repulsion parameter, $r_{ij}=\left|r_{i}-r_{j}\right|$, and ${\hat{r}}_{ij}=\left(r_{i}-r_{j}\right)/r_{ij}$. The dissipative and random forces are given by

(3) $$F_{ij}^{\left(D\right)}=-\gamma \;w{\left(r_{ij}\right)}^{2}\left({\hat{r}}_{ij}\cdot v_{ij}\right){\hat{r}}_{ij},\;F_{ij}^{\left(R\right)}=\zeta_{ij}\;w\left(r_{ij}\right)\sqrt{\frac{6k_{B}T\gamma }{\Delta t}}\;{\hat{r}}_{ij},$$

where γ is the friction coefficient, $v_{ij}=v_{i}-v_{j}$, $\zeta_{ij}$ is a uniformly distributed random number in [−1,1] generated independently for each interacting pair at each time step, and Δt is the integration time step. The weight function is chosen as

(4) $$w\left(r\right)=\left\{\begin{array}{cc} 1-\frac{r}{r_{c}}, & r<r_{c},\\ 0, & r\geq r_{c}. \end{array}\right.$$

Bond connectivity is imposed via a harmonic spring force,

(5) $$F_{ij}^{\left(S\right)}=-C\left(r_{ij}-r_{0}\right){\hat{r}}_{ij},$$

where *C* is the spring constant and $r_{0}$ is the equilibrium bond length. The fluctuation–dissipation balance between $F_{ij}^{\left(D\right)}$ and $F_{ij}^{\left(R\right)}$ maintains the system at the prescribed reduced temperature $k_{B}T$.

We used reduced DPD units with $r_{c}=1$, m=1, and $k_{B}T=1$. The equations of motion were integrated with a velocity-Verlet scheme using Δt=0.01. Simulations were performed in a cubic periodic box of side length $L_{\mathrm{box}}=40.0$ at bead number density ρ=3.0, corresponding to the melt state. The full set of DPD and architectural parameters used in this work is summarized in Table 1.

Two bead types (A and B) were used. The repulsion between identical bead types was fixed at $a_{AA}=a_{BB}=a_{ii}=25.0$, while the unlike repulsion was set to $a_{AB}=a_{ii}+\Delta a$, where Δa controls the segregation strength. The two segregation strengths considered in this work are $\Delta a/k_{B}T=4.1$ (WSR) and 24.5 (SSR). Using the commonly adopted mapping at ρ=3.0, $\Delta a/k_{B}T=3.27\;\chi$ [36], these correspond to χ≈1.25 and 7.5, i.e., $\chi N_{d}\approx 15$ and 90 for $N_{d}=12$. In addition, to generate equilibrated structures and phase maps, we scanned $\Delta a/k_{B}T$ stepwise from 0 to 24.5 in increments of 4.1.

We investigated two classes of systems (Figure 1). The first is a copolymacromer-like hybrid bottlebrush architecture, denoted as (AB)-g-A, where symmetric AB diblock side chains and A-type homopolymer side chains are grafted to a common backbone to form a single covalent macromolecule. The backbone length was fixed at M=16 beads, and each backbone bead carried exactly one side chain. A total of $M_{d}$ symmetric AB diblock side chains (each of total length $N_{d}$ with $N_{d}=N_{A}+N_{B}$ and $N_{A}=N_{B}=6$) and $M_{h}=16-M_{d}$ A-type homopolymer side chains (length $N_{h}$) were grafted to the backbone.

The key architectural control parameters are the side-chain length ratio $\alpha \equiv N_{h}/N_{d}$ and the homopolymer segment fraction $\phi \equiv \frac{M_{h}N_{h}}{M_{d}N_{d}+M_{h}N_{h}}$, defined based on side-chain segments. The second class is a composition-matched blend consisting of free symmetric AB diblock chains (length $N_{d}=12$ with $N_{A}=N_{B}=6$) and free A-type homopolymer chains (length $N_{h}$). For each (α,ϕ) considered in the copolymacromer system, the blend composition was constructed to match the diblock length, homopolymer length, and overall homopolymer segment fraction, thereby isolating the effect of covalent integration versus physical mixing.

All systems were initialized from random configurations and prepared using a staged equilibration protocol with a stepwise increase in segregation strength. After generating a random initial state, we equilibrated the melt at Δa=0 for $1.0\times 10^{6}$ steps. We then increased $\Delta a/k_{B}T$ stepwise from 0 to 24.5 in increments of 4.1; at each Δa, we performed $2.5\times 10^{6}$ equilibration steps followed by $5.0\times 10^{5}$ production steps, yielding $3.0\times 10^{6}$ steps per Δa stage.

Morphologies were identified from time-averaged coarse-grained concentration fields and classified into lamellae (L), bicontinuous morphologies (B), cylinders (C), micelles (S), and disordered states (D). For visualization of the self-assembled morphologies, the instantaneous particle configurations were coarse-grained onto a regular three-dimensional grid with a spacing of $1.0\;r_{c}$, corresponding to a 40×40×40 grid for the present simulation box ($L_{\mathrm{box}}=40$). The local volume fraction field $\phi_{i}\left(r\right)$ was computed by assigning beads to grid cells and normalizing by the cell volume. The concentration fields were averaged over the production stage ($5.0\times 10^{5}$ steps), with configurations sampled every $5\times 10^{3}$ steps. The rendered structures correspond to iso-surfaces of the time-averaged A-segment concentration field. For blends, macrophase-separated states were additionally labeled as two-phase (2) when large-scale demixing prevented assignment of a single periodic microdomain morphology. Two-phase coexistence in the blends (labeled “2”) was diagnosed from the phase relation of long-wavelength concentration fluctuations between the diblock species (d) and the homopolymer species (h), following the phase-correlation approach based on complex cross-correlations [38]. We computed the complex cross-correlation spectrum

(6) $$S_{d,h}\left(k\right)\equiv V^{-1}\langle {\hat{\psi }}_{d}\left(k\right)\;{\hat{\psi }}_{h}\left(-k\right)\rangle ,$$

where $\psi_{i}\left(r\right)=\phi_{i}\left(r\right)-\langle \phi_{i}\rangle$(i=d,h) is the concentration fluctuation and ${\hat{\psi }}_{i}\left(k\right)$ denotes its Fourier transform. The phase difference between the two concentration waves is quantified as

(7) cosΔφ(k)=ReSd,h(k)Sd,h(k),

where Δφ is the phase difference. In practice, the k→0 limit is probed by the smallest accessible nonzero wavevector $k_{\min}=2\pi /L_{\mathrm{box}}$. Here, the dominant low-*k* mode was identified as the wavevector that maximizes $|S_{d,h}\left(k\right)|$ within the low-*k* window. A blend state was labeled as two-phase when the dominant cross-correlation in the low-*k* region occurred at $k_{\min}$ and satisfied $\Delta \varphi (k_{\min})\approx \pi$ (i.e., cos(Δφ)≈−1), indicating anti-correlated, box-scale composition fluctuations consistent with macrophase demixing in the thermodynamic limit. For lamellar states, the domain spacing *L* was obtained from the period of the one-dimensional composition profile along the lamellar normal and normalized by $L_{0}$, the lamellar spacing at ϕ=0 under the same α and $\Delta a/k_{B}T$. Selective domain swelling was quantified by the lamellar width ratio $L_{A}/L_{B}$, where $L_{A}$ and $L_{B}$ are the thicknesses of A- and B-rich domains extracted from the same composition profile.

## 3. Results

**Figure 2:**
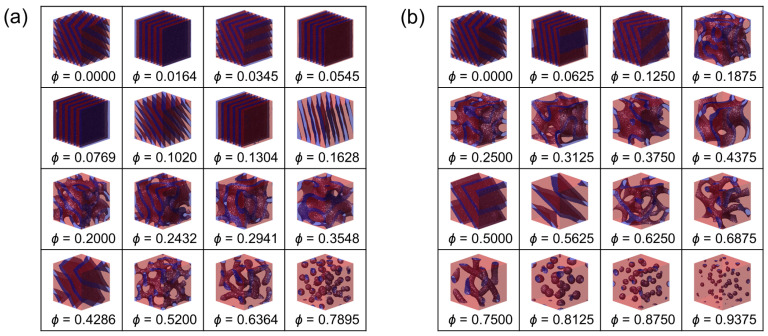
Representative DPD morphologies of CH-BBC melts at $\Delta a/k_{B}T=24.5$ for (**a**) α=0.25 and (**b**) α=1.0. Within each panel, the homopolymer segment fraction ϕ is varied as indicated beneath each morphology. A-rich and B-rich domains are rendered in red and blue, respectively.

**Figure 3:**
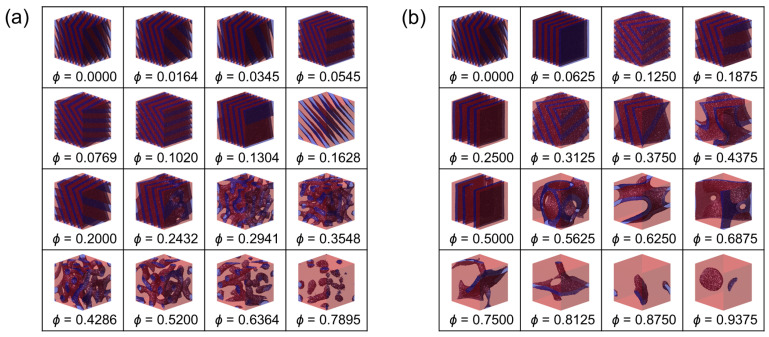
Representative DPD morphologies of composition-matched blends (AB + A) at $\Delta a/k_{B}T=24.5$ for (**a**) α=0.25 and (**b**) α=1.0. Within each panel, the homopolymer segment fraction ϕ is varied as indicated beneath each morphology. A-rich and B-rich domains are rendered in red and blue, respectively.

We begin by directly inspecting the self-assembled morphologies obtained under SSR, $\Delta a/k_{B}T=24.5$ (corresponding to $\chi N_{d}\approx 90$ for $N_{d}=12$), where microphase separation is well developed and architectural effects on ordering and swelling can be visually assessed. Figure 2 and Figure 3 show representative three-dimensional structures of (i) the copolymacromer-like hybrid bottlebrush (CH-BBC) and (ii) the composition-matched blend, respectively, for two side-chain length ratios, α=0.25 and 1.0, over a wide range of homopolymer segment fractions. The time-averaged concentration fields reveal systematic changes in domain connectivity, periodicity, and minority-domain morphology with increasing A-type homopolymer content.

For CH-BBC (Figure 2), the morphology evolves systematically with increasing ϕ in a manner consistent with progressive enrichment and swelling of the A-rich domain while maintaining a single, system-spanning microphase-separated state across the explored window. At low homopolymer loading, the structures are lamella-like, exhibiting alternating A- and B-rich layers that fill the simulation box. As ϕ increases, the lamellar pattern becomes increasingly distorted and transitions into network-like, bicontinuous morphologies in which the B-rich phase remains percolated through the A-rich matrix. Upon further addition of the A-type homopolymer, the B-rich domains lose connectivity and transform into discrete objects (cylinder-/micelle-like and ultimately sphere-like B-rich domains) dispersed within a continuous A-rich matrix at the highest ϕ. Importantly, throughout this progression the structures remain globally homogeneous in the sense that the microdomains occupy the full simulation volume without a macroscopic A-rich region separating from a distinct BCP-rich region. This observation is consistent with the architectural constraint that A-type homopolymer side chains are tethered to the same scaffold that contains B-forming segments, forcing the additional A content to be accommodated primarily by dilation and morphological reorganization of the existing microphase-separated framework rather than by macroscopic demixing.

The corresponding blend structures (Figure 3) share qualitative similarities with CH-BBC at low ϕ, where lamella-like ordering characteristic of symmetric AB diblocks is obtained for both α=0.25 and 1.0. With increasing ϕ, the blend also exhibits the expected progression toward more A-rich morphologies, including network-like states and ultimately isolated B-rich domains embedded in an A-rich matrix at high ϕ. However, in contrast to CH-BBC, the blend configurations display a more pronounced tendency toward large-scale compositional heterogeneity as ϕ increases, especially for the longer homopolymer case α=1.0. In this regime, B-rich domains become sparse and highly asymmetric in shape and distribution, and the global microstructure can lose the appearance of a single, uniformly periodic length scale across the box. Such features are consistent with the intuitive expectation for blends that free homopolymer chains can preferentially segregate into A-rich regions without the connectivity constraint imposed in CH-BBC, thereby enhancing composition fluctuations and facilitating pathways toward macrophase demixing when the homopolymer loading is sufficiently high.

Figure 2 and Figure 3 provide an immediate qualitative basis for the central premise of this work. Both systems exhibit the canonical sequence of microphase motifs driven by the underlying AB incompatibility, confirming that the diblock component dictates the formation of A/B interfaces under strong segregation. The key difference emerges in how additional A-type material is accommodated. In CH-BBC, covalent integration of the homopolymer into the microphase-forming scaffold channels the added A content into microdomain swelling and morphology transitions while preserving a single microphase-separated state. In blends, the absence of covalent linkage permits stronger large-scale composition fluctuations at elevated ϕ, leading to the emergence of two-phase coexistence and the consequent limitation of accessible microphase dilation, which we quantify and map in the following analyses.

**Figure 4:**
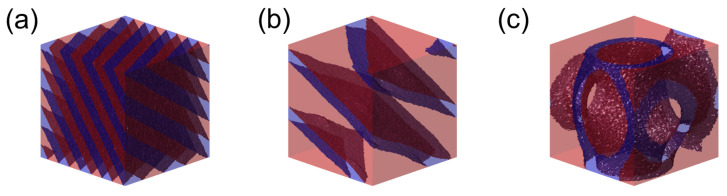
Lamellar dilation under the strongly segregated condition ($\Delta a/k_{B}T=24.5$, $\chi N_{d}\approx 90$ for $N_{d}=12$). (**a**) CH-BBC morphology at ϕ=0, showing the reference lamellar structure. (**b**) CH-BBC morphology at α=1 and ϕ=0.5625, illustrating preservation of lamellar topology with an enlarged lamellar period upon increasing homopolymer loading. (**c**) Composition-matched blend (AB + A) at α=1, ϕ=0.5625, and $\Delta a/k_{B}T=24.5$, showing destabilization of lamellar order under identical conditions. A-rich and B-rich domains are colored in red and blue, respectively.

Figure 4 provides a direct visual demonstration of lamellar dilation enabled by the CH-BBC architecture under the strongly segregated condition (SSR), $\Delta a/k_{B}T=24.5$ ($\chi N_{d}\approx 90$ for $N_{d}=12$). For CH-BBC, the reference melt at ϕ=0 forms well-defined lamellae with a uniform periodicity across the simulation box (Figure 4a). At a fixed side-chain length ratio α=1, increasing the homopolymer segment fraction to ϕ=0.5625 preserves the lamellar topology while enlarging the lamellar period and thickening the A-rich layers (Figure 4b), indicating that the additional A-type homopolymer content is accommodated primarily through swelling of the existing microdomains rather than by a morphology change that eliminates lamellar order. This behavior is consistent with the connectivity constraint in CH-BBC, where the A-type homopolymer is covalently integrated into the same macromolecular scaffold that also contains B-forming segments, thereby favoring internal redistribution and microdomain dilation over large-scale demixing pathways available to free blends.

In contrast, the composition-matched blend at the same α, ϕ, and $\Delta a/k_{B}T$ does not sustain a lamellar morphology (Figure 4c), highlighting that free homopolymer addition can destabilize the lamellar state at elevated loading under otherwise identical thermodynamic driving forces. The loss of a system-spanning lamellar pattern in the blend is consistent with enhanced long-wavelength composition fluctuations and the onset of two-phase coexistence.

To generalize the qualitative trends observed in Figure 2, Figure 3 and Figure 4 and to identify the parameter window in which microphase dilation is accessible, we constructed phase diagrams as functions of the homopolymer segment fraction ϕ and the side-chain length ratio α.

**Figure 5:**
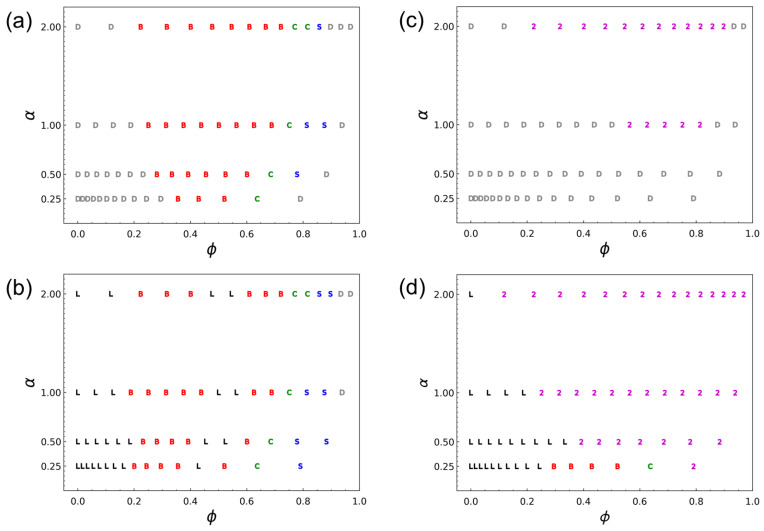
Phase diagrams in the (ϕ,α) plane comparing CH-BBC melts and composition-matched blends at two segregation strengths. (**a**,**b**) CH-BBC melts: (**a**) weak-segregation regime (WSR), $\Delta a/k_{B}T=4.1$ ($\chi N_{d}\approx 15$ for $N_{d}=12$), and (**b**) strong-segregation regime (SSR), $\Delta a/k_{B}T=24.5$ ($\chi N_{d}\approx 90$ for $N_{d}=12$). (**c**,**d**) Composition-matched blends (AB + A): (**c**) WSR, $\Delta a/k_{B}T=4.1$, and (**d**) SSR, $\Delta a/k_{B}T=24.5$. Here, ϕ denotes the homopolymer segment fraction and $\alpha \equiv N_{h}/N_{d}$ is the side-chain length ratio. The symbols indicate the observed states: L (lamellae), B (3D bicontinuous), C (cylinders), S (micelles), D (disordered), and 2 (two-phase coexistence/macrophase-separated states in blends).

Figure 5 summarizes the resulting morphology maps at two segregation strengths: the weak-segregation regime (WSR), $\Delta a/k_{B}T=4.1$ ($\chi N_{d}\approx 15$), and the strong-segregation regime (SSR), $\Delta a/k_{B}T=24.5$ ($\chi N_{d}\approx 90$). For CH-BBC (Figure 5a,b), the phase behavior is described solely by microphase morphologies (L/B/C/S) and the disordered state (D) because the AB diblock and A homopolymer segments are covalently integrated within a single macromolecular species; thus, macrophase demixing into AB-rich and A-rich phases (the two-phase state in blends) is excluded by construction. In the WSR (Figure 5a), a large portion of the explored parameter space remains disordered, as expected near the order–disorder boundary for short symmetric diblocks, while ordered microphases emerge over intermediate ϕ and become more prevalent as α increases. In the SSR (Figure 5b), ordered states dominate over a broad region. Overall, lamellae appear at low ϕ, bicontinuous morphologies occupy much of the intermediate-ϕ window, and cylinders/micelles emerge as ϕ increases further, consistent with an effective increase in A-rich content induced by incorporation of A-type homopolymer side chains. Notably, this progression is not strictly monotonic: at selected α values, a reentrant lamellar window appears between two bicontinuous regions, yielding the sequence L–B–L–B–C with increasing ϕ. This reentrant lamellar region can be understood as the result of two competing effects of increasing ϕ. On the one hand, increasing ϕ enhances the overall A-rich asymmetry, which tends to favor morphologies with nonzero interfacial curvature. On the other hand, because the A-type homopolymer is tethered to the same scaffold as the diblock side chains, the added A-type material must be accommodated within the existing microphase-separated framework rather than behaving as a free additive. As quantified below, the tethered homopolymer preferentially fills and expands the A-domain interior, whereas the diblock A segments remain localized near the interfaces. This architecture-specific redistribution can re-stabilize a swollen lamellar state over an intermediate composition window, producing the reentrant L region. At still larger ϕ, the overall A/B asymmetry again dominates, and the system returns to interfacially curved morphologies, leading to the subsequent B and C regions.

The blend phase behavior (Figure 5c,d) differs qualitatively by exhibiting extensive two-phase coexistence (labeled “2”). Here, “2” denotes two-phase coexistence in the blends identified by the low-*k* (k→0) out-of-phase criterion, $\Delta \phi (k_{\min})\approx \pi$, as described in the Simulation Methods; representative visual examples of this state are shown in the high-ϕ region of Figure 3b and in Figure 4c. In the WSR (Figure 5c), the blend is largely disordered, and two-phase states already appear at sufficiently large α and moderate-to-high ϕ, indicating that macrophase demixing can preempt ordered microphase formation when the homopolymer is long under weak segregation. In the SSR (Figure 5d), lamellae persist at low ϕ for all α, but the two-phase region expands rapidly with increasing ϕ and α, severely restricting the composition window over which a single microphase morphology can be maintained in the blend. This direct comparison shows that covalent integration in CH-BBC replaces the blend’s two-phase region with an extended microphase-separated window, thereby enabling microphase dilation at elevated homopolymer loading. In the following, we quantify the extent of dilation and selective domain swelling using the normalized domain spacing $L/L_{0}$ and the lamellar width ratio $L_{A}/L_{B}$.

**Figure 6:**
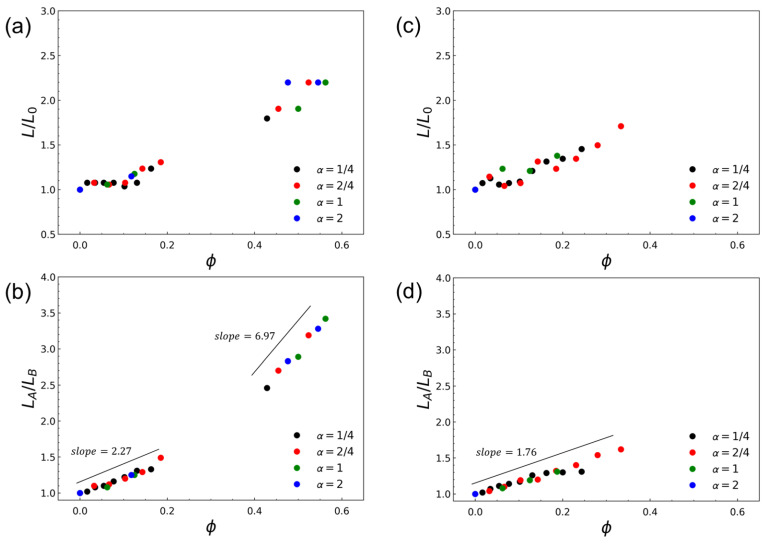
Quantification of lamellar dilation and selective domain swelling under the strongly segregated condition (SSR), $\Delta a/k_{B}T=24.5$ ($\chi N_{d}\approx 90$ for $N_{d}=12$). (**a**,**c**) Normalized lamellar period, $L/L_{0}$, as a function of the homopolymer segment fraction ϕ for (**a**) CH-BBC and (**c**) the composition-matched blend (AB + A). (**b**,**d**) Lamellar width ratio, $L_{A}/L_{B}$, as a function of ϕ for (**b**) CH-BBC and (**d**) the blend, where $L_{A}$ and $L_{B}$ denote the thicknesses of A- and B-rich domains, respectively. Data points are shown only for conditions identified as lamellae. $L_{0}$ denotes the lamellar period at ϕ=0 for the corresponding system. Colors indicate the side-chain length ratio $\alpha \equiv N_{h}/N_{d}$. The straight lines indicate linear fits (or guides to the eye); for CH-BBC two regimes with slopes of 2.27 (low ϕ) and 6.97 (high ϕ) are shown, whereas for the blend a single slope of 1.76 is shown. The homopolymer segment fraction is denoted by ϕ in the plots.

Figure 6 quantifies microphase dilation within the lamellar state under the strongly segregated condition ($\Delta a/k_{B}T=24.5$, $\chi N_{d}\approx 90$), enabling a direct comparison between CH-BBC and the composition-matched blend beyond the qualitative morphological analysis presented above. For CH-BBC (Figure 6a), the normalized lamellar period $L/L_{0}$ increases modestly at low homopolymer loading (ϕ≲0.2) but exhibits a pronounced increase at higher ϕ, reaching $L/L_{0}\approx 2.2$ for α≳0.5 in the accessible lamellar window. In contrast, the blend (Figure 6c) shows only a gradual increase in $L/L_{0}$ with ϕ and attains a substantially smaller dilation (up to $L/L_{0}∼1.7$) before lamellae cease to be observed over much of the parameter space, consistent with the expansion of the two-phase region in the blend phase diagram.

The lamellar width ratio further demonstrates that dilation in CH-BBC is strongly selective for the A-rich domain. As shown in Figure 6b, $L_{A}/L_{B}$ for CH-BBC displays a clear crossover from a weak increase at low ϕ (slope ≈2.27) to a steep growth regime at higher ϕ (slope ≈6.97), indicating that additional A-type homopolymer content is incorporated predominantly by thickening the A domain rather than by a symmetric expansion of both domains. By comparison, the blend exhibits a much weaker, nearly single-regime increase in $L_{A}/L_{B}$ with ϕ (slope ≈1.76; Figure 6d), implying limited selective swelling within the lamellar state. Together, these trends show that covalent integration of A-type homopolymer side chains enables substantially larger lamellar dilation and stronger A-domain swelling than is attainable in composition-matched blends, where the lamellar window is restricted by macrophase demixing and loss of lamellar stability at elevated homopolymer loading. This quantitative contrast motivates the density-based analyses presented below, which clarify how tethered homopolymer segments and diblock segments partition across the A-rich domain during dilation.

**Figure 7:**
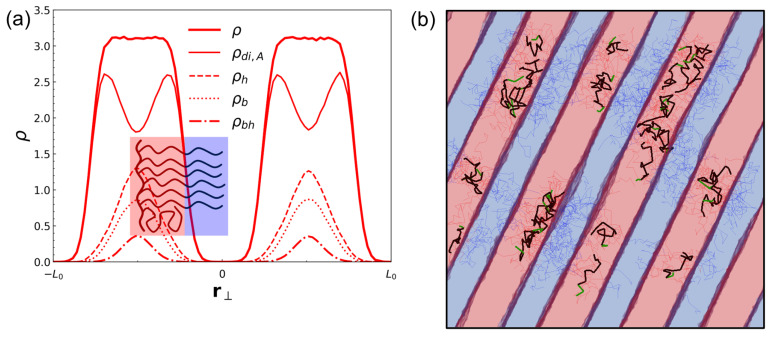
Density decomposition across lamellae for CH-BBC under strong segregation (SSR). (**a**) One-dimensional number-density profiles of A-type segments along the direction normal to the lamellar interfaces, $r_{⊥}$, for CH-BBC at ϕ=0.125, α=1, and $\Delta a/k_{B}T=24.5$. The total A-segment density is denoted by ρ and is decomposed into contributions from A segments belonging to the diblock side chains ($\rho_{di,A}$), the A-type homopolymer side chains ($\rho_{h}$), the A-type backbone segments ($\rho_{b}$), and the subset of backbone segments connected to homopolymer side chains ($\rho_{bh}$). (**b**) Representative snapshot of molecular conformations in the corresponding lamellar morphology. Thick black segments indicate backbone bonds between grafting sites connected to diblock side chains, whereas thick green segments indicate backbone bonds connected to homopolymer side chains; thin red and blue segments represent A- and B-type side-chain bonds, respectively. The homopolymer segment fraction is denoted by ϕ.

To elucidate the molecular mechanism underlying lamellar dilation in CH-BBC, we analyzed the spatial distribution of A-type segments across the lamellar normal and decomposed the A-segment density under the SSR ($\Delta a/k_{B}T=24.5$) into contributions from diblock A segments ($\rho_{di,A}$), homopolymer side-chain segments ($\rho_{h}$), total A-type backbone segments ($\rho_{b}$), and the subset of backbone segments connected to homopolymer side chains ($\rho_{bh}$). At low homopolymer loading (ϕ=0.125; Figure 7a), the total A-segment density forms the expected A-rich plateau, while the decomposed profiles reveal a non-uniform partitioning within the A domain: $\rho_{di,A}$ is enhanced near the A/B interfaces, whereas $\rho_{h}$ is centered more toward the interior of the A-rich layer. This separation of roles is consistent with the grafted architecture, where diblock side chains maintain junction-driven proximity to interfaces to sustain well-defined A/B boundaries, while the tethered A-type homopolymer side chains preferentially occupy the domain interior.

**Figure 8:**
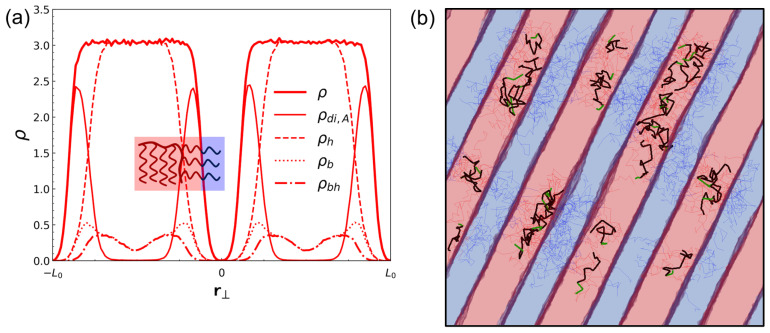
Redistribution of A-type segments in dilated lamellae for CH-BBC under strong segregation (SSR). (**a**) One-dimensional number-density profiles of A-type segments along $r_{⊥}$ for CH-BBC at ϕ=0.5625, α=1, and $\Delta a/k_{B}T=24.5$, shown with the same decomposition as in Figure 7a. (**b**) Representative snapshot of molecular conformations in the corresponding lamellar morphology, using the same rendering convention as in Figure 7b. The homopolymer segment fraction is denoted by ϕ.

At higher homopolymer loading corresponding to dilated lamellae (ϕ=0.5625; Figure 8a), the redistribution becomes more pronounced. The homopolymer contribution $\rho_{h}$ broadens and effectively fills the A-domain interior, whereas $\rho_{di,A}$ becomes increasingly localized near the interfacial regions. This evolution provides a direct mechanistic basis for the strong, selective swelling of the A domain quantified by the steep increase in $L_{A}/L_{B}$ at high ϕ in CH-BBC: additional A-type material is incorporated primarily by filling and expanding the A-rich domain interior, while the diblock A segments continue to stabilize the interfaces required for lamellar order. The corresponding snapshots (Figure 7b and Figure 8b) further support this picture by showing that backbone segments associated with diblock grafts remain closely aligned with interfacial regions, while backbone segments associated with homopolymer grafts populate the swollen A-rich layers. Overall, the density decomposition demonstrates that CH-BBC realizes microphase dilation through an internal, tethered filling mechanism that preserves microphase connectivity and interface integrity, rationalizing the substantially larger dilation window relative to composition-matched blends.

## 4. Conclusions

Covalent integration of A-type homopolymer side chains into a microphase-forming scaffold fundamentally alters phase behavior relative to conventional blends. Using DPD simulations, we compared a copolymacromer-like hybrid bottlebrush architecture, (AB)-g-A (CH-BBC), with composition-matched blends of free symmetric AB diblocks and free A homopolymers. Phase behavior was mapped as a function of the homopolymer segment fraction ϕ and side-chain length ratio $\alpha \equiv N_{h}/N_{d}$ under weak- and strong-segregation conditions, $\Delta a/k_{B}T=4.1$ (WSR; $\chi N_{d}\approx 15$) and $\Delta a/k_{B}T=24.5$ (SSR; $\chi N_{d}\approx 90$).

The resulting phase diagrams demonstrate a qualitative architectural effect. In blends, a broad two-phase coexistence region develops at elevated ϕ and/or larger α, indicating that macrophase demixing can preempt or destabilize ordered microphases. In contrast, CH-BBC is a one-component melt by construction, and the blend-like macrophase demixing pathway is removed; the accessible states are described by microphase morphologies and disorder. Under the SSR, CH-BBC exhibits an extended microphase-separated window across wide ranges of (ϕ,α), enabling systematic morphology evolution with increasing A-type content without the large two-phase region that limits blends.

Within the lamellar regime, CH-BBC enables substantially larger and more selective microphase dilation. The normalized lamellar period increases up to $L/L_{0}\approx 2.2$ for CH-BBC, whereas the blend shows a smaller increase and loses lamellar stability over much of the parameter space. Selective swelling is captured by the lamellar width ratio $L_{A}/L_{B}$, which grows sharply in CH-BBC at higher ϕ, indicating that added A-type material predominantly expands the A-rich domain rather than symmetrically dilating both domains. Density decompositions across lamellae reveal the molecular origin of this behavior: A segments on diblock side chains remain preferentially localized near A/B interfaces, stabilizing interfacial structure, while tethered homopolymer A segments fill and broaden the A-domain interior. This internal redistribution of tethered homopolymer segments provides a mechanistic basis for large lamellar dilation while preserving lamellar order.

The present results show that copolymacromer-like covalent hybridization can replace blend-limited macrophase demixing with a broad microphase-separated regime, thereby expanding the accessible dilation window and increasing the attainable domain spacing at fixed segregation strength. These insights suggest practical architectural routes to tune microdomain dimensions without sacrificing morphological integrity, and they motivate future studies addressing polydispersity in grafting/side-chain lengths, larger molecular sizes, and experimental realizations of (AB)-g-A copolymacromers for dilation-controlled nanostructured materials.

## Figures and Tables

**Table 1:** DPD parameters and architectural parameters used in this work. Reduced DPD units are employed, with the basic units defined as $r_{c}=1$ (length), m=1 (mass), and $k_{B}T=1$ (energy).

Meaning	Symbol	Value
Simulation box length	$L_{\mathrm{box}}$	40.0
Bead number density	ρ	3.0
Reduced temperature	$k_{B}T$	1.0
Cutoff radius	$r_{c}$	1.0
Time step	Δt	0.01
Friction coefficient	γ	4.5
Like-bead repulsion	$a_{ii}$ ($a_{AA}=a_{BB}$)	25.0
Spring constant	*C*	100.0
Bond rest length	$r_{0}$	0.7
Backbone length (CH-BBC)	*M*	16
Diblock side-chain length	$N_{d}$	12 ($N_{A}=N_{B}=6$)
Homopolymer side-chain length	$N_{h}$	3, 6, 12, 24
Number of diblock side chains (CH-BBC)	$M_{d}$	1, 2, …, 16
Number of homopolymer side chains (CH-BBC)	$M_{h}$	$16-M_{d}$

## Data Availability

The data presented in this study are available on request from the corresponding author.
